# Parents’ Perceptions of Student Academic Motivation During the COVID-19 Lockdown: A Cross-Country Comparison

**DOI:** 10.3389/fpsyg.2020.592670

**Published:** 2020-12-18

**Authors:** Sonia Zaccoletti, Ana Camacho, Nadine Correia, Cecília Aguiar, Lucia Mason, Rui A. Alves, João R. Daniel

**Affiliations:** ^1^Department of Developmental and Socialization Psychology, University of Padova, Padua, Italy; ^2^Faculty of Psychology and Education Sciences, University of Porto, Porto, Portugal; ^3^School of Health, Polytechnic of Porto, Porto, Portugal; ^4^Instituto Universitário de Lisboa (ISCTE-IUL), CIS-IUL, Lisbon, Portugal; ^5^William James Center for Research, ISPA–Instituto Universitário, Lisbon, Portugal

**Keywords:** COVID-19 pandemic, home confinement, school closures, distance learning, academic motivation, extracurricular activities, parents’ perceptions, cross-country comparison

## Abstract

The COVID-19 outbreak has ravaged all societal domains, including education. Home confinement, school closures, and distance learning impacted students, teachers, and parents’ lives worldwide. In this study, we aimed to examine the impact of COVID-19-related restrictions on Italian and Portuguese students’ academic motivation as well as investigate the possible buffering role of extracurricular activities. Following a retrospective pretest–posttest design, 567 parents (*n*_*Italy*_ = 173, *n*_*Portugal*_ = 394) reported on their children’s academic motivation and participation in extracurricular activities (grades 1 to 9). We used a multi-group latent change score model to compare Italian and Portuguese students’: (1) pre-COVID mean motivation scores; (2) rate of change in motivation; (3) individual variation in the rate of change in motivation; and (4) dependence of the rate of change on initial motivation scores. Estimates of latent change score models showed a decrease in students’ motivation both in Italy and in Portugal, although more pronounced in Italian students. Results also indicated that the decrease in students’ participation in extracurricular activities was associated with changes in academic motivation (i.e., students with a lower decrease in participation in extracurricular activities had also a lower decrease in motivation). Furthermore, students’ age was significantly associated with changes in motivation (i.e., older students had lower decrease). No significant associations were found for students’ gender nor for parents’ education. This study provides an important contribution to the study of students’ academic motivation during home confinement, school closures, and distance learning as restrictive measures adopted to contain a worldwide health emergency. We contend that teachers need to adopt motivation-enhancing practices as means to prevent the decline in academic motivation during exceptional situations.

## Introduction

The COVID-19 pandemic has changed everyday life, imposing unprecedented sanitary, political, economic, social, and educational challenges. Home confinement and temporary school closures have also affected educational systems worldwide (Reimers and Schleicher, 2020; World Bank Group, 2020). The transition to distance learning placed a heavy strain on teachers, students, and their parents (Cachón-Zagalaz et al., 2020; Hiraoka and Tomoda, 2020). The rapid shift in the delivery mode of instruction and an uncertain future may have led students to experience considerable challenges in maintaining their academic motivation. However, to the best of our knowledge, although some evidence exists, for instance, on student’s learning habits during COVID-19 (Trung et al., 2020), there is no empirical evidence specifically on how the COVID-19 pandemic and the restrictive measures, adopted by national governments to contain the new coronavirus spread, impacted students’ academic motivation and learning across countries.

In this study, we surveyed Italian and Portuguese parents during April and May of 2020 to examine the impact of the COVID-19 pandemic and the associated restrictive measures on their children’s academic motivation. Additionally, we also aimed to investigate the association between changes in academic motivation and students’ participation in extracurricular activities during the COVID-19 lockdown to get a broader sense of the impacts of the imposed restrictive measures.

We analyzed data from these countries since they share similar culture and values regarding family functions in society and show similar patterns of relations and obligations between teachers and students (Hofstede, 2001; Pepi et al., 2006). However, the new coronavirus struck Italy long before Portugal, which was able to adopt restrictive measures within a short period of time. In effect, as reported by the European Centre for Disease Prevention and Control (2020) on 1st of April 2020 in Italy there were 4053 cases and 839 deaths, while in Portugal on the same date there were 1035 cases and 20 deaths.

In the next sections we present: (a) an overview of the educational contexts in Italy and Portugal; (b) available evidence on the impact of COVID-19 on education; and (c) available literature on the role of student’s academic motivation and extracurricular activities on their academic performance.

### Educational Contexts in Italy and Portugal

In Italy, compulsory education starts at the age of 6 and ends at the age of 16 and includes three cycles of education: (1) five years of primary school (i.e., grades 1 to 5); (2) three years of lower secondary school (i.e., grades 6 to 8); and (3) two years of upper secondary school (i.e., grades 9 and 10). Students can then decide to complete upper secondary school (i.e., grades 11 to grade 13) (EACEA/EURYDICE, 2019). In Portugal, compulsory education starts at the age of 6 and ends at the age of 18, and encompasses nine years of basic education (grades 1–9) and three years of secondary education (grades 10–12). Basic education consists of nine years of schooling divided into three sequential cycles of four (i.e., 1st cycle - grades 1 to 4), two (i.e., 2nd cycle - grades 5 and 6), and three years (i.e., 3rd cycle - grades 7 to 9) (EACEA/EURYDICE, 2019).

### The Impact of COVID-19 Pandemic on Education

The COVID-19 pandemic affected every societal domain, including educational systems worldwide. Among the restrictive measures imposed to contain the spread of the new coronavirus (SARS-CoV-2), home confinement was one of the most severe measures that national governments adopted worldwide. This measure has led to an exceptional and temporary suspension of teaching activities attendance. As of late April 2020, 85% of students worldwide were out of school due to school closures in 180 countries (World Bank Group, 2020).

In the most affected areas of Italy, schools and universities suspended academic activities on February 24th, followed by a nationwide lockdown from March 9th onwards (Decree-Law 23 febbraio 2020 no. 6, 2020). As the new coronavirus spread across Europe, other national governments undertook similar measures. In Portugal, schools closed from March 16th onwards (Decree-Law no. 14/2020 86(89)-86(19), 2020), although many families voluntarily self-isolated from the beginning of March. While in Italy schools closed 16 days after the 50th confirmed COVID-19 case, in Portugal schools closed only four days after reaching the same threshold (Santana et al., 2020).

During school closures, teachers adopted new strategies, such as distance learning programs and open educational applications and platforms, to reduce disruption and ensure that students could receive instruction remotely. In this regard, parental support became essential, especially for younger students, who are not fully autonomous in managing the assigned learning activities. Nevertheless, these strategies raised concerns as not all parents could work alongside their children, nor every household had the required electronic devices such as laptops with wi-fi connection (Reimers and Schleicher, 2020). Furthermore, it is well known that school and home are two separate environments that require students to play different roles. In this situation, however, students have found themselves without physical contact either with their teachers nor with their classmates.

Critically, if some students may find motivation to engage in school activities as a challenge in itself, the COVID-19 pandemic and the associated restrictive measures (i.e., home confinement, school closures, and distance learning) may have further hindered students’ ability to sustain academic motivation towards school activities, such as attending online and asynchronous classes, studying, and doing homework.

### Academic Motivation

Motivation is a catalyst of human behavior. Schunk and Pintrich (2002, p. 5) defined motivation as “the process whereby goal-directed activity is instigated and sustained.” This definition highlights that motivation is shaped and constrained by both contextual factors and individual characteristics (Anderman and Dawson, 2011). Motivation is not an unidimensional concept; instead it is a complex, multidimensional construct that encompasses different components, such as beliefs, goals, values, desires, needs, and emotions (Murphy and Alexander, 2000; Wentzel and Wigfield, 2009; Anderman and Dawson, 2011).

Across the past four decades, researchers have aimed to understand how motivation enhances students’ learning and achievement in school. Academic motivation pertains to students’ beliefs, goals, and values that determine which academic or school-related tasks they will pursue and persist in Wentzel and Wigfield (2009). According to Gottfried (1990, p. 525), academic motivation is the “enjoyment of school learning characterized by a mastery orientation; curiosity; persistence; task-endogeny; and the learning of challenging, difficult, and novel tasks.”

Given the multidimensional nature of motivation, researchers have proposed different theories of achievement motivation (e.g., self-determination theory, social cognitive theory, self-theories, expectancy-value theory). Despite this profusion of motivation-related theories, there is a consensus among researchers that academic motivation is associated with positive academic and health-related skills and outcomes, such as self-regulation, persistence, critical thinking, academic achievement, school completion, career success, psychological well-being, and physical health (e.g., Guay et al., 2008; Archambault et al., 2009; Lai, 2011; De Naeghel et al., 2012; Cerasoli et al., 2014; Kriegbaum et al., 2015; Lazowski and Hulleman, 2016; Yilmaz Soylu et al., 2017; Camacho et al., 2020).

Within the context of the self-determination theory (Deci and Ryan, 1985; Ryan and Deci, 2000), students tend to become more intrinsically motivated when they experience satisfaction of the psychological needs for competence, autonomy, and relatedness in a learning task or context. *Competence* refers to the need of being effective in one’s pursuits and interactions with the social environment (Deci, 1975). Students’ need for competence is fulfilled when they know how to effectively achieve outcomes (e.g., through rules, feedback) (Skinner and Belmont, 1993). For instance, receiving positive feedback on a task fulfills students’ need for competence, thus increasing intrinsic motivation. *Autonomy* refers to the perception of being causal agents of one’s own life. Zuckerman et al. (1978) found that intrinsic motivation increased when individuals have options and choices to deal with when performing a task. On the contrary, external factors that restrict the perception of control, like deadlines, lead to a decrement of intrinsic motivation. Finally, *relatedness* refers to the importance of being emotionally connected and in interaction with other people. Fulfillment of the need for relatedness is likely to occur when teachers and peers create an authentic, caring, and supportive environment. When these three psychological needs are not fulfilled, students may experience maladjustment and lack of motivation.

Previous research also indicated that academic motivation typically declines over the school years. This decline seems to be consistent across grade-levels and across several motivational constructs (Lazowski and Hulleman, 2016; Scherrer and Preckel, 2019). Given that academic motivation boosts students’ learning and achievement, drops in motivation are troublesome. Therefore, researchers need to determine which instructional practices bolster academic motivation and then translate these findings into evidence-based practices for teaching purposes. In this regard, a meta-analytic review by Lazowski and Hulleman (2016) showed that motivation interventions produced robust effects on behavioral educational outcomes and academic performance across diverse samples. For instance, within the context of the self-determination theory, motivation interventions include brief activities or instructional practices, such as allowing students to perceive more freedom and choice in learning activities, thus fulfilling the psychological need for autonomy.

Prior research has also shed light on gender-based differences in academic motivation. That is, gender differences in motivation seem to be domain specific as boys tend to report more favorable motivational beliefs in mathematics, science, and sports, while girls usually report more positive motivational beliefs in language, arts, reading, and writing (Meece et al., 2006). However, more recent evidence showed that girls reported higher levels of general academic motivation (Bugler et al., 2015). Both parents and teachers can contribute to gender differences in academic motivation by implicitly modeling gender-typed behaviors and by conveying different expectations, goals, and tasks for girls and boys (Meece et al., 2006).

Parents also actively influence student academic motivation. According to the self-determination theory, the fulfillment of students’ psychological needs for competence, autonomy, and relatedness also depend on: (a) the way parents organize the environment (i.e., definitions of rules, guidelines, and expectations); (b) parent autonomy-support and respect for children’s perspectives; and (c) the extent to which parents are involved and establish positive, caring relationships with their children (Grolnick et al., 2009). In addition, researchers have relied on reports from significant adults, namely parents and teachers, as reliable sources to assess student motivation and academic performance (e.g., Gilger, 1992; Harris et al., 2006; Saçkes et al., 2016; Allerhand, 2018; Owens et al., 2020).

Importantly, despite the wealth of research on academic motivation, there is scant evidence on whether and how extreme situations—such as home confinement, school closures, and distance learning as restrictive measures adopted in response to a global health emergency—may hamper students’ motivation as perceived by their parents.

### Extracurricular Activities

Participation in extracurricular activities, which applies to all levels of education, has received increased attention in research (Massoni, 2011). Extracurricular activities are optional activities, physically or mentally stimulating, encompassing structure, and providing experiences not included in formal academic or study activities (Larson and Verma, 1999; Massoni, 2011). They might refer to sports, clubs, student councils, or other activities that generally appeal to student interests, and encourage peer interaction, promote cooperation, strengthen student-adult relationships, and provide structure and challenge (Van Etten et al., 2008; Massoni, 2011).

Given the relevance of extracurricular activities, extracurricular settings are considered important contexts for development, and students’ transactions within those settings are described as proximal drivers of development (Fredricks and Simpkins, 2013). In effect, participating in structured extracurricular activities is associated with positive outcomes for students (Gilman et al., 2004). For instance, research suggests that student participation in extracurricular activities has been positively associated with academic performance and school connectedness (Holloway, 2002; Akos, 2006). Further, students participating in structured activities supervised by adults are more likely to invest in their schooling, becoming more motivated to excel academically (Jordan and Nettles, 1999). In this regard, several studies have documented positive associations between participation in extracurricular activities and students’ academic motivation (Fredricks and Simpkins, 2013; Im et al., 2016).

In addition, participating in extracurricular activities mitigates behavioral risks. High-risk students (e.g., from disadvantaged backgrounds) involved in school extracurricular programs are less likely to drop out of school and to be involved in errant (e.g., delinquent) behaviors (Mahoney, 2000), while becoming academically more motivated (Mahoney et al., 2005; Im et al., 2016). Likewise, participation in extracurricular activities is described as a relevant protective factor impacting both achievement and psychosocial adjustment, contributing to foster more successful school transition experiences (Akos, 2006).

Research is inconsistent regarding the effect of students’ gender on their participation in extracurricular activities. Indeed, although some authors suggest that boys and girls are equally likely to take part in extracurricular activities (Holloway, 2002; Guèvremont et al., 2008), others suggest that girls are either more or less likely to engage in this type of activities (Darling et al., 2005; Newman et al., 2007). Regarding age, overall, older students have been shown to have higher participation rates than younger ones (Guèvremont et al., 2008).

Available evidence also suggests that students whose parents have lower education levels are less likely to participate in extracurricular activities than students from more highly educated families (Darling et al., 2005). In addition, previous research has pointed to the lack of parent understanding of the academic benefits of extracurricular activities, and their consequent lack of support, as barriers to students’ participation in extracurricular activities (Im et al., 2016).

Further, previous studies have addressed the role extracurricular activities can play (e.g., in reducing deviant behavior, or augmenting positive adjustment of students) in exceptional circumstances, such as managing racial conflicts (Crain, 1981), coordinating emergency crises, such as a mass school disaster (Klingman, 1987), or even promoting medical students’ learning during a war (Gluncic et al., 2001). However, to the best of our knowledge, no studies have tackled their role and their associations with students’ academic motivation, during a worldwide health emergency. Nonetheless, existing studies highlighted the beneficial effects of extracurricular activities for students’ well-being, and their potential role as a buffer for academic motivation.

## The Present Study

The main purpose of this study was to examine the impact of COVID-19 restrictions (i.e., home confinement, school closures, and distance learning) on students’ academic motivation and the extent of their participation in extracurricular activities. Adopting an exploratory approach, we compared students’ academic motivation in Italy and Portugal—two Southern European, culturally similar countries (Hofstede, 2001; Pepi et al., 2006) that faced the spread of COVID-19 at different times.

We invited Italian and Portuguese parents to report their perceptions of their children’s academic motivation and participation in extracurricular activities for two time periods: (1) before the onset of COVID-19; and (2) during the COVID-19 lockdown period. We turned to parents and not directly to students due to two reasons. On the one hand, using online data collection procedures, it was difficult to reach children due to ethical and time constraints. On the other hand, we wanted to focus on students from grades 1 to 9 and young students may find it challenging to reflect on their academic motivation, especially by means of a self-report scale. We thus relied on parents’ reports given that previous research has demonstrated the relevance and accuracy of parents’ report measures (e.g., Gilger, 1992; Saçkes et al., 2016; Allerhand, 2018; Owens et al., 2020).

Specifically, two research questions (RQ) guided the study: (1) Did students’ academic motivation decrease from the period before the onset of COVID-19 (hereafter, pre-COVID-19) to the COVID-19 lockdown period (hereafter, COVID-19)?; (2) Were changes in students’ participation in extracurricular activities associated with changes in academic motivation from the pre-COVID-19 to the COVID-19 period?

For RQ1, we expected to find a decrease in students’ academic motivation during the COVID-19 lockdown period given the challenges posed by lockdown measures in both countries. That is, our first hypothesis was that home confinement, school closures, and distance learning threatened the fulfillment of the psychological needs for competence, autonomy and relatedness, which are linked to intrinsic motivation (Deci and Ryan, 1985; Ryan and Deci, 2000).

For RQ2, since previous studies have demonstrated the positive relation between participation in extracurricular activities and students’ academic motivation (Fredricks and Simpkins, 2013; Im et al., 2016) we expected changes in the extent of participation in extracurricular activities to be positively associated with changes in academic motivation in both countries. That is, a putative decrease in academic motivation (between pre-COVID-19 and COVID-19) would be associated with a decrease in extracurricular activities.

In our statistical analyses, we included three important demographic variables: students’ age and gender, and parents’ education. First, we considered students’ age since younger students might have found it harder to cope with lockdown measures and maintain their academic motivation, given their evolving cognitive, metacognitive, and self-regulation abilities (Zimmerman, 1990; Dignath et al., 2008). In addition, younger students generally need more support from their parents when dealing with academic assignments, because they are less autonomous than older students.

Second, we took potential gender differences into account as previous studies have shown differences both in academic motivation and participation in extracurricular activities between girls and boys (e.g., Darling et al., 2005; Bugler et al., 2015). We also took parents’ education into account as this is a common control variable in educational studies that has been shown to relate with students’ academic achievement and participation in extracurricular activities (Eccles, 2005; van Tetering et al., 2018).

## Materials and Methods

### Participants

This study was approved by the Ethical Committee for the Psychological Research of the University of Padova (authorization number: 3530). We safeguarded participants’ data privacy in compliance with the General Data Protection Regulation (GDPR).

A convenience sample of 567 parents from different regions in Italy (*n* = 173, 89% mothers) and Portugal (*n* = 394, 93% mothers) participated in this study. Participants were recruited via social media platforms (e.g., Facebook). After their consent to participate in the study, participants completed an online survey. Mean parent age was 42.90 (*SD* = 5.76) for the Italian sample and 41.00 (*SD* = 5.53) for the Portuguese sample. As for educational level, 83% of parents in the Italian sample reported to have completed at least high school, compared to 89% in the Portuguese sample. Concerning income, 50% of Italian parents reported at least a 30,000€ yearly income, compared to 10,000€ of Portuguese parents.

Students’ mean age was 9.65 (*SD* = 2.14; 46% girls) for the Italian sample and 10.04 (*SD* = 2.52; 52% girls) for the Portuguese sample. In the Italian sample, 72% of students were in primary school (grades 1 to 5), whereas 28% were in lower secondary school (grades 6 to 8). In the Portuguese sample, 50% of the students were in the first cycle of basic education (grades 1 to 4), 34% were in the second cycle of basic education (grades 5 to 6), and 16% were in the third cycle of basic education (grades 7 to 9).

### Measures

#### Academic Motivation

Parents completed a questionnaire reporting their perceptions about their children’s academic motivation before the onset of COVID-19 (pre-COVID-19) and in the lockdown period (COVID-19). From an initial pool of 12 items, adapted from an Italian standardized test—AMOS 8–15 (Cornoldi et al., 2005)—we selected 5 to index academic motivation (see Supplementary Material for further details on item selection). Two examples of items were: “When the teacher assigns homework, my child does it by self-initiative and not because parents ask her/him to” and “My child studies the minimum to get a sufficient grade”. Items were the same both at pre-COVID-19 and COVID-19 but with different instructions. For the former, parents had to think about children’s academic motivation during a routine situation before the onset of the COVID-19 pandemic; for the latter, parents had to think about children’s academic motivation during the lockdown period. Items were scored from 1 (*I completely disagree*) to 5 (*I completely agree*) and were highly reliable both at pre-COVID-19 (McDonald’s ωt_*Italy*_ = 0.84, McDonald’s ωt_*Portugal*_ = 0.88) and at COVID-19 time points (McDonald’s ωt_*Italy*_ = 0.86, McDonald’s ωt_*Portugal*_ = 0.89).

#### Participation in Extracurricular Activities

To assess students’ participation in extracurricular activities (i.e., as perceived by parents), Italian and Portuguese parents were asked a single question: “How many hours does the child practice extracurricular activities during the week?” The response scale ranged from 1 to 5 (1 = *does not practice any*; 2 = *1–2 h*, 3 = *3–4 h*, 4 = *5–6 h*, 5 = *more than 6 h a week*). Parents answered the question bearing in mind the period before COVID-19 (pre-COVID-19: *M*_*Italy*_ = 3.19, *SD*_*Italy*_ = 1.03 and *M*_*Portugal*_ = 2.88, *SD*_*Portugal*_ = 1.17) and the COVID-19 lockdown period (COVID-19: *M*_*Italy*_ = 1.12, *SD*_*Italy*_ = 0.57; Portugal: *M*_*Portugal*_ = 1.29, *SD*_*Portugal*_ = 0.69).

### Procedure

**FIGURE 1:**
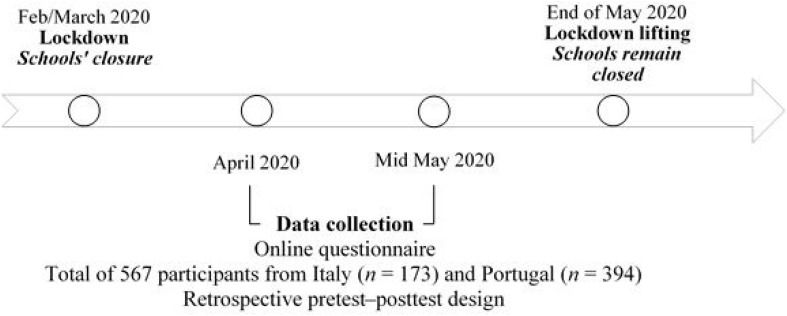
Timeline of lockdown, school closures, and data collection in Italy and Portugal.

After providing online consent, Italian and Portuguese parents completed the online survey through the Qualtrics XM Platform (Qualtrics, 2019). Data collection took place from April (i.e., during the COVID-19 lockdown) until the middle of May 2020 (i.e., before the lockdown was lifted) in both countries. Parents were recruited using official university channels and social media networks (e.g., Facebook, LinkedIn). We used a retrospective pretest–posttest design (Little et al., 2020). Figure 1 depicts the restrictive measures and the data collection timeline in Italy and Portugal.

### Data Analysis Approach

First, to answer our RQ1 we used a multi-group latent change score model (LCSM; Kievit et al., 2018) to compare motivation changes across time and samples. The four parameters of interest of the LCSM are the: (1) pre-COVID mean latent motivation score; (2) mean latent change score (i.e., the rate of change in motivation); (3) latent change score variance (i.e., individual variation in the rate of change in motivation); and the (4) covariance between pre-COVID-19 motivation and the mean latent change score (i.e., the dependence of rate of change on initial motivation scores). All measurement model parameters were constrained to be equal across time and sample (see Supplementary Table 3). Following Kievit et al. (2018), we fitted a set of models, sequentially constraining these four parameters. Likelihood-ratio tests were used to test for significant differences across samples.

Next, to answer our RQ2 we computed a multi-group bivariate LCSM to test the association between the rate of change in academic motivation and the rate of change in extracurricular activities. This corresponds to combining a LCSM of extra-curricular activities to the model estimated above, plus a covariance parameter between motivation and extracurricular activities rates of change.

Finally, we tested whether the rates of change of motivation and extracurricular activities were associated with three demographic variables (viz., children’s gender, children’s age, and parents’ education levels).

All LCSMs were estimated using the lavaan package (version 0.6-5; Rosseel, 2012) in R (version 3.6.1; R Core Team, 2019).

## Results

Results are presented in the following order: (1) descriptive statistics for the motivational scale and participation in extracurricular activities item; (2) academic motivation latent change score model; (3) academic motivation and participation in extracurricular activities bivariate latent change score model; and (4) final model comprising the association between rates of change in academic motivation and participation in extracurricular activities with socio-demographic variables.

### Descriptive Statistics

Table 1 summarizes descriptive statistics for academic motivation and extent of participation in extracurricular activities across Italian and Portuguese samples. In both countries, academic motivation and participation in extracurricular activities mean values decreased in the COVID-19 lockdown period.

**TABLE 1:** Mean Scores for Pre and Post-COVID Motivation (M1–M5) and Extra-Curricular (EC) Activities.

	pre-COVID-19		COVID-19	
		
	*M*	*SD*	*M*	*SD*
**Italy (*N* = 173)**				
M1	3.09	1.10	2.79	1.10
M2	3.08	1.06	2.82	1.10
M3	3.20	1.13	2.87	1.13
M4	3.70	1.11	3.42	1.06
M5	3.47	1.09	3.22	1.09
EC activities	3.19	1.03	1.12	0.57
**Portugal (*N* = 394)**				
M1	3.10	1.24	3.01	1.25
M2	3.28	1.22	3.21	1.18
M3	3.19	1.19	2.99	1.15
M4	3.46	1.23	3.24	1.17
M5	3.42	1.15	3.26	1.16
EC activities	2.88	1.17	1.29	0.69

*All items’ scores ranged from 1 to 5.*

### Academic Motivation Latent Change Score Model

**FIGURE 2:**
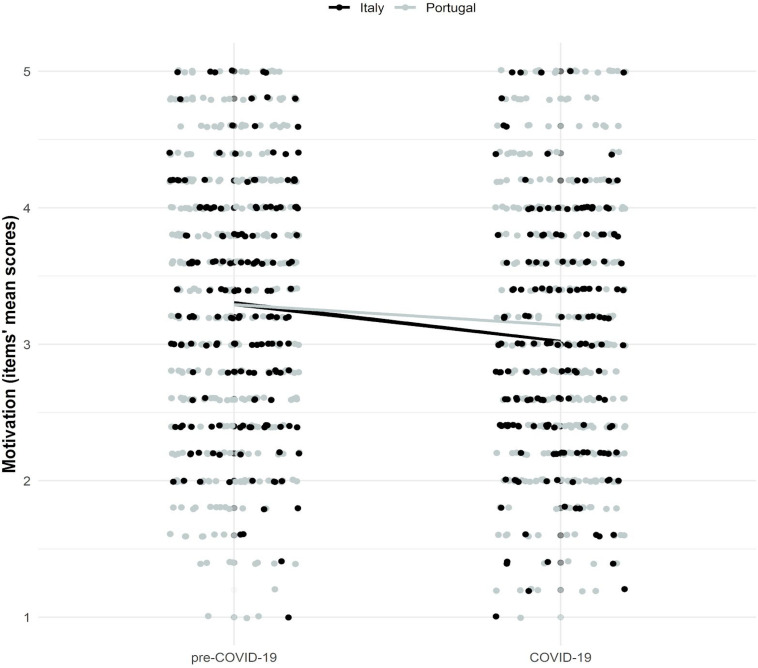
Motivation changes across time and samples. Pre-COVID-19 motivation items’ mean scores (*M*_*Italy*_ = 3.31, *M*_*Portugal*_ = 3.29) are similar to the pre-COVID-19 mean latent motivation score estimated by the LCSM (3.10). COVID-19 motivation items’ mean decrease (Italy = −0.29, Portugal = −0.15) is similar to the mean latent change score estimated by the LCSM (Italy = −0.27, Portugal = −0.14; *p* < 0.001). Figure drawn using ggplot2 package (3.2.1; Wickham, 2016) in R.

Model estimates showed that Portuguese and Italian students had similar initial levels of motivation (pre-COVID-19 mean latent motivation score = 3.10; likelihood ratio test: *χ*^2^_(1)_ difference = 0.00, *p* = 0.950; see Figure 2). Importantly, model estimates indicated significant decreases in motivation (mean latent change score: Italy = −0.27, *p* < 0.001; Portugal = −0.14, *p* < 0.001), particularly in the Italian sample (likelihood ratio test: χ^2^_(1)_ difference = 6.67, *p* = 0.010). We also found significant individual variability in the rates of change (latent change score variance = 0.36, *p* < 0.001), but this variability was similar across samples (likelihood ratio test: χ^2^*_(1)_* difference = 1.27, *p* = 0.260). Finally, decreases in motivation were significantly higher for students with higher pre-COVID-19 motivation scores (co-variance = −0.17, *p* < 0.001), and this association was similar across samples [likelihood ratio test: χ^2^_(1)_ difference = 0.16, *p* = 0.690].

Approximate fit indexes of the motivation latent change score model, with unconstrained mean latent change scores (i.e., different rate of change across samples), showed acceptable to good values (robust measures - χ2_(105)_ = 254.61, *p* < 0.001; CFI = 0.96, TLI = 0.96, RMSEA = 0.08 [0.06, 0.09], SRMR = 0.07, Yuan-Bentler scaling factor = 1.13).

### Academic Motivation and Participation in Extracurricular Activities Bivariate Latent Change Score Model

Similarly to the previous section, we fitted a set of models, sequentially constraining the following parameters: (1) pre-COVID-19 mean extracurricular activities; (2) extracurricular activities mean latent change score; (3) latent change score variance; and (4) pre-COVID-19 participation in extracurricular activities and mean latent change score covariance.

**FIGURE 3:**
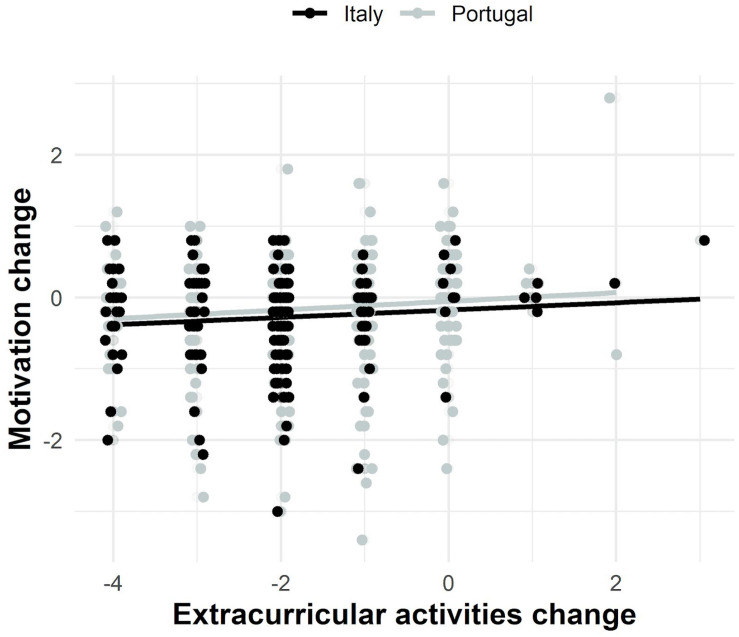
Association between academic motivation and extracurricular activities change across samples. Motivation (extracurricular activities) change equals the difference between COVID-19 mean motivation items’ scores (extracurricular activities score) and pre-COVID-19 mean motivation items’ scores (extracurricular activities score). The positive regression lines approximate the positive and significant association between motivation and extracurricular activities mean latent change scores estimated by the bivariate LCSM for both samples (co-variance = 0.04, *p* = 0.030). Figure drawn using ggplot2 package (3.2.1; Wickham, 2016) in R.

Model estimates show that the mean level of participation in extracurricular activities pre-COVID-19 differed across samples [likelihood ratio test: χ^2^_(1)_ difference = 10.41, *p* = 0.001], with higher levels of participation in extracurricular activities for the Italian students (estimated mean extracurricular activities: Italy = 3.19, Portugal = 2.88; see Figure 3). Model estimates further showed significant decreases in extracurricular activities (mean latent change scores: Italy = −2.08, *p* < 0.001; Portugal = −1.59, *p* < 0.001), particularly in Italy [likelihood ratio test: χ^2^_(1)_ difference = 13.76, *p* < 0.001].

Results also indicated significant individual differences in the rates of change (latent change score variance = 1.51, *p* < 0.001), but that this variability was similar across samples (likelihood ratio test: χ^2^_(1)_ difference = 0.06, *p* = 0.813).

Similarly to academic motivation, decreases in the level of participation in extracurricular activities were significantly higher for students with higher pre-COVID levels of participation in extracurricular activities (co-variance = −1.17, *p* < 0.001), and this association was similar across samples [likelihood ratio test: χ^2^_(1)_ difference = 0.37, *p* = 0.546].

Most notably, the rate of change in motivation and the rate of change in extracurricular activities were positively and significantly associated (co-variance = 0.04, *p* = 0.030). This association was similar across samples [likelihood ratio test: χ^2^_(1)_ difference = 1.52, *p* = 0.217].

Approximate fit indexes of the motivation latent change score model, with unconstrained pre-COVID-19 mean levels of extracurricular activities and unconstrained mean latent change scores, showed acceptable to good values [robust measures- χ^2^_(146)_ = 306.78, *p* < 0.001; CFI = 0.95, TLI = 0.96, RMSEA = 0.07 [0.05, 0.08], SRMR = 0.07, Yuan-Bentler scaling factor = 1.09].

### Final Model With Socio-Demographic Variables

For the final set of models, we tested whether the rates of change of motivation and extracurricular activities were associated with students’ gender (covariate: girls = −0.5, boys = 0.5), students’ age, and parents’ education levels. As such, we added 6 co-variances to the previous model. Next, we tested whether these associations were equal across samples using likelihood ratio tests. Finally, we dropped non-significant variables.

**FIGURE 4:**
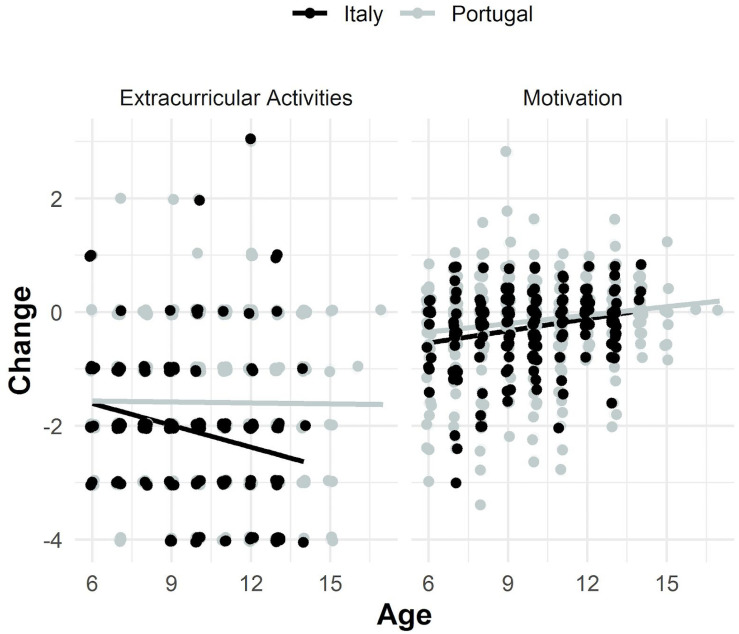
Association between age and motivation and extracurricular activities change across samples. Motivation change equals the difference between post-COVID mean motivation items’ scores and pre-COVID mean motivation items’ scores. Extracurricular activities change equals the difference between post-COVID reported extracurricular activities levels and pre-COVID reported extracurricular activities levels. The bivariate LCSM estimated that for both samples children’s age was positively and significantly associated with the motivation mean latent change score (co-variance = 0.31, *p* < 0.001), but not with the extra-curricular activities mean latent change score (co-variance = 0.10, *p* = 0.101). Figure drawn using ggplot2 package (3.2.1; Wickham, 2016) in R.

Model estimates showed that the association between socio-demographic variables and the rates of change were similar across samples [likelihood ratio test: χ^2^_(7)_ difference = 1.94, *p* = 0.963]. Students’ age was positively and significantly associated with the rate of change in motivation (co-variance = 0.31, *p* < 0.001), with older students showing lower decreases in motivation, but not with the participation in extracurricular activities rate of change (co-variance = 0.10, *p* = 0.101; see Figure 4). However, students’ gender and parents’ education levels were not associated with either changes in motivation, or in the participation in extracurricular activities.

**FIGURE 5:**
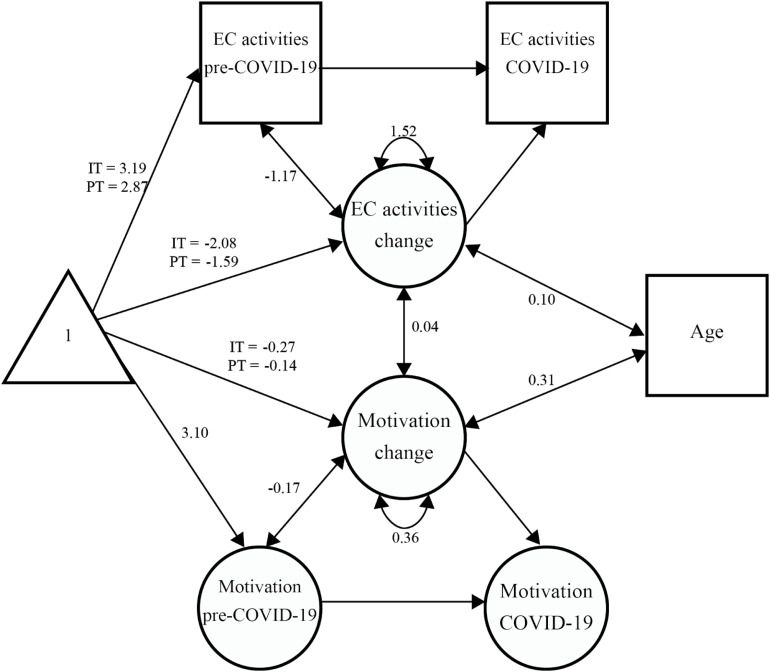
Unstandardized final model estimates. For simplicity, only estimates of interest of the bivariate LCSM are presented. Shapes follow standard figural notation for structural equation modeling: triangle - intercepts (estimate mean levels), squares - manifest/observed variables, circles - latent variables, one-headed arrows - unidirectional effects (regression weights or means), and double-headed arrows - (co-) variances. EC = Extracurricular, IT = Italy, PT = Portugal.

Approximate fit indexes of the final model showed acceptable to good values [robust measures: χ^2^_(168)_ = 369.59, *p* < 0.001; CFI = 0.94, TLI = 0.95, RMSEA = 0.07 [0.06, 0.08], SRMR = 0.07, Yuan-Bentler scaling factor = 1.08]. Figure 5 depicts relevant unstandardized model estimates (see Supplementary Table 4 for complete unstandardized model estimates).

## Discussion

The main purpose of this study was to examine the impact of COVID-19 restrictions (i.e., home confinement, school closures, and distance learning) on students’ academic motivation and participation in extracurricular activities, as perceived by their parents. We sampled parents from two European countries that are culturally similar but that had to deal with the virus outbreak at different times. Therefore, we conducted a retrospective pretest–posttest study, where Italian and Portuguese parents reported their perceptions pertaining to children’s academic motivation and participation in extracurricular activities before the onset of the COVID-19 pandemic and during the lockdown period.

Our first hypothesis was confirmed since we found a decrease in students’ academic motivation during the lockdown period, as perceived by parents. In this regard, the self-determination theory (Deci and Ryan, 1985; Ryan and Deci, 2000) may be especially useful to interpret a drop in students’ academic motivation. As aforementioned, according to this theory, student engagement in school is influenced by the degree to which they perceive that the school context meets their psychological needs. Home confinement, school closures, and distance learning may have threatened the satisfaction of competence, autonomy, and relatedness needs, thereby hindering students’ academic motivation. In this regard, we put forth three possible interpretations for our findings.

First, we suggest that the situation of uncertainty, at times without clear instructions and expectations from teachers, led to the need for competence not being fulfilled. Indeed, students need clear tasks, feedback, deadlines, as well as individualized support, and activities with appropriate levels of difficulty. Moreover, teachers had fewer opportunities to become aware of individual students’ emotional and academic difficulties. This may have further impacted students’ perceptions of competence.

Second, students rapidly had to become more autonomous in organizing their time and completing tasks assigned by teachers. Therefore, younger children may have had more difficulty managing themselves and a more substantial need for support from parents, who have diverse levels of preparedness and availability to assist their children in school matters. While the satisfaction of the need for autonomy is fundamental to promote intrinsic motivation, it is important to underline that the ability to self-manage develops over time. Therefore, younger students may not have fully developed this ability. Moreover, it has been demonstrated that younger students are more likely to be affected by contextual factors (Sameroff, 2010). Older students’ self-regulation strategies are more developed, which probably allowed them to cope more adaptively with the emergency situation and to preserve - albeit to a limited extent - their academic motivation.

Third, during lockdown, students’ opportunities to interact with teachers and peers directly were drastically reduced, likely resulting in decreases in connectedness. In this regard, it has been widely demonstrated that the need for relatedness is likely to occur when teachers and peers create supportive relationships. Indeed, teacher support has been positively related to indicators of behavioral engagement, including higher participation in school activities and academic motivation and fewer disruptive behaviors (e.g., Wang et al., 2013). Moreover, previous research has demonstrated that students who have positive interactions with peers are more motivated in engaging in school activities (Wang and Eccles, 2013).

Our second hypothesis was also supported. In line with previous research (e.g., Fredricks and Simpkins, 2013; Im et al., 2016), we found a positive and significant association between the rate of change in extracurricular activities and the rate of change in academic motivation. These findings are also in line with research suggesting that motivation in the school context (i.e., academic motivation) is associated with students’ involvement in other contexts, such as leisure-time activities (Vallerand and Ratelle, 2002). Therefore, it seems that being involved in extracurricular activities is important to promote motivation in the school context (Wallhead et al., 2010). These findings are particularly relevant, as they highlight the important buffering role that extracurricular activities may have on students’ academic motivation, also during exceptional times. Moreover, although research on participation in extracurricular activities has focused largely on high school students, our findings inform about the role of extracurricular activities in younger students. These years are particularly important, as students are undergoing rapid biological, cognitive, and social changes, such as the increased exposure to peers and the increased desire for autonomy (Im et al., 2016).

In this study, we adopted an exploratory approach in comparing students’ academic motivation changes across Italy and Portugal. Interestingly, we found that students’ decrease in academic motivation was higher for Italian than Portuguese students. In Italy, the first two official cases of COVID-19 were recorded between the end of January and the beginning of February of 2020, while in Portugal that only happened in the beginning of March (STATISTA, 2020). In particular, Italy was an European hotspot of the pandemic, which forced the government to adopt restrictive measures gradually, such as isolating specific geographical areas, closing schools and universities, limiting social interactions, restricting people and travel movement, and ceasing non-essential activities temporarily (Gatto et al., 2020; Sebastiani et al., 2020). Whereas in Portugal, national authorities had the chance to adopt the same restrictive measures early on (Santana et al., 2020), which curbed the new coronavirus spread during the first wave of infections. Therefore, the COVID-19 pandemic may have imposed a heavier psychological toll on the Italian population, including students who were called to remain focused and motivated in school activities (away from their teachers and classmates) in a situation of dramatic health emergency.

### Limitations

This study has some limitations that open the door for future research. First, we conducted a retrospective pretest–posttest study (Little et al., 2020), rather than a longitudinal study. Therefore, conclusions drawn about the trajectory of academic motivation before and during COVID-19 lockdown period should be interpreted with caution. Future longitudinal studies could shed more light on the trajectory of students’ academic motivation and participation in extracurricular activities during an exceptional situation, such as the COVID-19 pandemic. Researchers could thus understand whether a decline in both academic motivation and participation in extracurricular activities endures or reverts to baseline levels over time, as well as disentangle causality effects.

Second, we examined parents’ perceptions rather than children’s perceptions of academic motivation. We opted to rely on parents’ reports mainly due to ethical issues involving children’s participation in online questionnaires. However, as aforementioned, previous research has demonstrated that parents are relevant (Dinkelmann and Buff, 2016) and accurate (Eccles, 2005) sources, for instance in reporting their perception on students’ literacy motivation (e.g., Saçkes et al., 2016). Future research could rely on children’s perceptions of their own academic motivation and participation in extracurricular activities.

Third, our sample is non-representative of Italian and Portuguese populations. Indeed, parents’ demographic characteristics were not equally distributed. We received overwhelmingly more answers from mothers than fathers. Additionally, parents portrayed in our study reported high levels of education and lived mainly in urban areas. Notwithstanding, we exerted the best of our efforts to disseminate the online study to parents from different Italian and Portuguese regions.

Fourth, other potentially relevant variables could have been taken into account in order to shed more light on the role of academic motivation and participation in extracurricular activities, such as contextual determinants (e.g., parental support during distance learning and teaching practices) and academic and psychological outcomes (e.g., academic achievement, self-regulation, and psychological well-being).

### Implications for Practice

Despite these limitations, results of this study may suggest relevant practical implications. We propose the need for the adoption of motivation-enhancing practices to thwart the decline in students’ academic motivation during exceptional situations, such as the COVID-19 lockdown. Teachers can nurture students’ motivation by using several practices that aim to satisfy the psychological needs for competence, autonomy, and relatedness (Deci and Ryan, 1985; Ryan and Deci, 2000). For instance, teachers may: (a) allow student choice and autonomy in the learning process; (b) introduce online collaborative learning methods; (c) establish rules, tasks, deadlines, and a learning environment that conveys the idea of the school structure; and (d) give students appropriate feedback. This way, students can maintain their academic motivation by having a more concrete idea of current and future expectations. For teachers, this is certainly a great challenge, as they are asked to promote students’ motivation through distance learning that shifts away from the more traditional forms of teaching, that is, through direct in-person contact.

Teachers and parents can also play a relevant role in promoting students’ participation in extracurricular activities, within the limits allowed by the restrictive measures (e.g., music lessons, drama clubs, dance practice). Prior research suggested that when encouraged to participate in extracurricular activities by a nurturing adult, students tend to put forth more effort into those activities, aiming to achieve higher levels of success and recognition (Hanline, 2018). Therefore, teachers, through distance contact, and parents, through direct contact, may encourage and support students in participating in extracurricular activities.

### Conclusion

This study provided insights into the changes in students’ academic motivation and participation in extracurricular activities, as perceived by their parents, during a dramatic historical period that affected the whole world. A key priority of education institutions is to promote and ensure students’ learning within a safe, supportive, and motivating environment. The spread of COVID-19 has led to new modalities of instruction to enable students to continue learning in a safe environment, which, during lockdown, was the student’s home. Therefore, this exceptional situation represented an inevitable challenge that required constant adaptation by teachers, students, and their parents. Despite the efforts of teachers and parents to mitigate the effects of the COVID-19 restrictive measures on students, our results showed a decrease in students’ academic motivation associated with a decrease in their participation in extracurricular activities.

In light of possible future exceptional situations, teachers and parents could combine their efforts to fulfill students’ psychological needs for competence, autonomy, and relatedness, thereby fostering their motivation. Moreover, they may encourage students’ participation in extracurricular activities to achieve a good balance between academic and extracurricular activities, acting as a buffer for academic motivation.

## Data Availability Statement

The raw data supporting the conclusions of this article will be made available by the authors, without undue reservation.

## Ethics Statement

The studies involving human participants were reviewed and approved by Ethical Committee for the Psychological Research, University of Padova, Italy. The patients/participants provided their written informed consent to participate in this study.

## Author Contributions

SZ performed conceptualization, methodology, writing original draft, review and editing, and project administration. AC performed conceptualization, methodology, writing original draft, and review and editing. NC performed methodology, writing original draft, and review and editing. CA performed supervision and writing review and editing. LM performed supervision and writing review and editing. RA performed supervision and writing review and editing. JD performed data analysis, writing original draft, review and editing. All authors contributed to the article and approved the submitted version.

## Conflict of Interest

The reviewer JG declared a shared affiliation with several of the authors. AC and RA to the handling editor at the time of review. The remaining authors declare that the research was conducted in the absence of any commercial or financial relationships that could be construed as a potential conflict of interest.
