# Supplementary material for: Parents’ Perceptions of Student Academic Motivation During the COVID-19 Lockdown: A Cross-Country Comparison
Source: Front Psychol. 2020 Dec 18;11:592670. doi: 10.3389/fpsyg.2020.592670 (PMC7775314; doi:10.3389/fpsyg.2020.592670)
Supplement: Supplementary file 1 [file Data_Sheet_1.PDF]

Supplementary Material

Parents' Perceptions of Student Academic Motivation during the COVID-19 Lockdown: A Cross-country Comparison

1. SUPPLEMENTARY FIGURES AND TABLES

1.1. Exploratory Factor Analysis (EFA)

We started by plotting scree plots (Cattell, 1966) to test whether the motivation items indexed a unidimensional construct, for both the pre-COVID-19 and COVID-19 item forms (Figure S1, Table S1). The scree plots indicated that one factor should be retained for EFA (Figure S1).

a) Pre-COVID-19 **b) COVID-19**

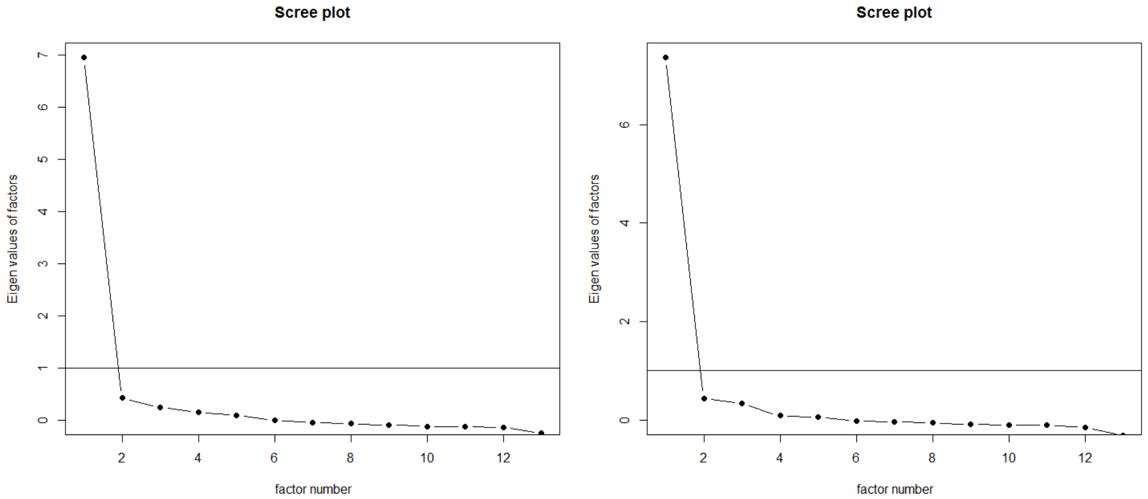

Figure S1. Scree plot of pre-COVID-19 and COVID-19 motivation items. Figures drawn using psych package (version 1.8.12; Revelle, 2019) in R.

Table S1. Pre-COVID-19 and COVID-19 motivation items' correlation matrix

	**M1**	**M2**	**M3**	**M4**	**M5**	**M6**	**M7**	**M8**	**M9**	**M10**	**M11**	**M12**
**Pre-COVID-19**												
M2	0.14											
M3	0.54	0.18										
M4	0.55	0.23	0.56									
M5	0.53	0.2	0.57	0.62								
M6	0.43	0.25	0.47	0.51	0.6							
M7	0.33	0.25	0.46	0.42	0.46	0.63						
M8	0.53	0.19	0.6	0.65	0.83	0.57	0.44					
M9	0.48	0.22	0.53	0.55	0.75	0.49	0.43	0.8				
M10	0.47	0.22	0.54	0.56	0.74	0.54	0.45	0.82	0.79			
M11	0.55	0.26	0.61	0.7	0.71	0.55	0.49	0.74	0.67	0.72		
M12	0.38	0.15	0.43	0.54	0.62	0.49	0.39	0.62	0.52	0.54	0.62	
M13	0.43	0.25	0.51	0.55	0.59	0.48	0.48	0.61	0.55	0.57	0.66	0.56
**COVID-19**												
M2	0.12											
M3	0.66	0.09										
M4	0.70	0.18	0.68									
M5	0.64	0.16	0.59	0.73								
M6	0.49	0.16	0.5	0.55	0.57							
M7	0.35	0.16	0.45	0.39	0.43	0.64						
M8	0.67	0.21	0.62	0.74	0.83	0.58	0.42					
M9	0.64	0.2	0.59	0.69	0.8	0.56	0.41	0.89				
M10	0.65	0.21	0.61	0.72	0.81	0.61	0.43	0.89	0.86			
M11	0.71	0.18	0.7	0.83	0.76	0.55	0.44	0.78	0.74	0.76		
M12	0.44	0.12	0.43	0.55	0.52	0.53	0.34	0.52	0.49	0.54	0.54	
M13	0.52	0.19	0.51	0.57	0.51	0.45	0.47	0.56	0.53	0.54	0.61	0.48

EFAs, using psych package (version 1.8.12; Revelle, 2019) in R, with one factor showed that, except for item M2, all items had strong loadings (Table S2). As such, item M2 was dropped from further analysis. Because several items had very similar wordings and were highly correlated we used a combination of factor loadings and items' discrimination (mean difference of the top third scores and lowest third scores) to decide which items to keep/drop. Within each set we selected one item. Selected items had the highest loadings and discrimination scores (excepting for M6 which only had higher discrimination in the COVID-19 EFA). Hereafter, the five selected items will be referred as M1 to M5 (both in the supplementary material file and in the main text).

Table S2. Pre-COVID-19 and COVID-19 motivation items' factor loadings and discrimination scores

	N	*M*	*SD*	Descrimination	Factor loading	Set	Selected	New item name
**Pre-COVID-19**								
M1	567	3.1	1.2	2.48	0.62	0	yes	M1
M2	567	3.16	1.08	1.81	0.28		no	
M3	567	2.76	1.18	2.03	0.70	1	no	
M4	567	3.45	1.2	2.11	0.75	1	no	
M5	567	3.28	1.17	2.05	0.86	2	no	
M6	567	3.54	1.2	2.15	0.69	3	yes	M4
M7	567	3.03	1.18	2.63	0.59	3	no	
M8	567	3.19	1.17	2.46	0.89	2	yes	M3
M9	567	2.83	1.12	1.96	0.81	2	no	
M10	567	3.05	1.11	2.38	0.83	2	no	
M11	567	3.22	1.17	2.5	0.86	1	yes	M2
M12	567	3.72	1.02	0.91	0.69	4	no	
M13	567	3.43	1.14	1.98	0.72	4	yes	M5
**COVID-19**								
M1	567	2.94	1.21	2.53	0.76	0	yes	M1
M2	567	3.06	1.06	2.36	0.21		no	
M3	567	2.74	1.16	2.01	0.74	1	no	
M4	567	3.21	1.15	1.99	0.85	1	no	
M5	567	3.11	1.11	2.4	0.86	2	no	
M6	567	3.29	1.14	2	0.69	3	yes	M4
M7	567	2.94	1.11	1.89	0.54	3	no	
M8	567	2.95	1.14	2.42	0.91	2	yes	M3
M9	567	2.79	1.13	1.97	0.87	2	no	
M10	567	2.93	1.15	2.42	0.89	2	no	
M11	567	3.09	1.17	2.47	0.89	1	yes	M2
M12	567	3.56	1.04	1.87	0.62	4	no	
M13	567	3.25	1.14	1.99	0.66	4	yes	M5

Note. Discrimination scores correspond to the mean difference of the top third scores (highest scores) and lowest third scores (lowest scores); items from the same set have similar wordings; final items' wording: M1- "*When it is time to do homework/study s/he is poorly motivated*" (reversed), M2- "*It is hard to convince him/her to do homework/study because s/he would rather perform other activities*" (reversed), M3- "*It is difficult to get him/her concentrate when s/he has to do homework/study*" (reversed), M4- "*S/he studies the minimum to get a sufficient grade (reversed)*", M5- "*When the teacher assigns homework, s/he does it all (by his own and not solicited by parents)*".

1.2. Longitudinal Confirmatory Factor Analysis (CFA)

Following Widaman, Ferrer, and Conger (2010) we tested the factorial invariance of motivation items across time and samples by comparing a series of increasingly restricted models (Table S3). According to Cheung and Rensvold (2002) a value of ΔCFI between 0 and –0.01 indicates invariance.

Table S3. Motivation factorial invariance

	CFI	⊗CFI
Configurational invariance	0.977	
Invariant factor loadings	0.975	-0.002
Invariant factor loadings and intercepts	0.959	-0.016
Invariant factor loadings, intercepts, and error variances	0.951	-0.008
Invariant factor loadings, intercepts, error variances and error co-variances	0.951	0.000

Table S3 results indicate that at least one of the motivation items is not invariant across time and/or sample. Comparing intercept invariance for one item at a time showed that two items' intercepts were non-invariant. Because multi-group, longitudinal models with a more restricted factorial invariance, that still maintain acceptable fit indices, have more interpretative value than less restricted models (despite their lower statistical fit), we opted to present in the main text models with full factorial invariance (Widaman et al., 2010). Nevertheless, the final model presented in the main text (complete estimates presented in Table S4) showed the same patterns of significance than a partial factorial invariance model where the invariance constraints of the two intercepts were relaxed.

1.3. Latent Change Score Model Estimates

Table S4. Unstandardized model estimates

	**Invariant**			**Italy**	
	estimate	*SE*	*p*	estimate	*SE*
**1. Factor loadings**					
1.1 Motivation items					
Motivation pre-COVID-19 =~ M1	1				
Motivation pre-COVID-19 =~ M2	1.22	0.05	< 0.001		
Motivation pre-COVID-19 =~ M3	1.18	0.05	< 0.001		
Motivation pre-COVID-19 =~ M4	0.91	0.05	< 0.001		
Motivation pre-COVID-19 =~ M5	0.91	0.05	< 0.001		
Motivation COVID-19 =~ M1	1				
Motivation COVID-19 =~ M2	1.22	0.05	< 0.001		
Motivation COVID-19 =~ M3	1.18	0.05	< 0.001		
Motivation COVID-19 =~ M4	0.91	0.05	< 0.001		
Motivation COVID-19 =~ M5	0.91	0.05	< 0.001		
1.2. Change					
Motivation change =~ Motivation COVID-19	1				
EC Activities change =~ EC Activities COVID-19	1				
**2. Intercepts**					
2.1. Motivation items					
M1 pre-COVID-19	0				
M2 pre-COVID-19	-0.52	0.15	< 0.001		
M3 pre-COVID-19	-0.49	0.15	0.001		
M4 pre-COVID-19	0.66	0.16	< 0.001		
M5 pre-COVID-19	0.59	0.16	< 0.001		
M1 COVID-19	0				
M2 COVID-19	-0.52	0.15	< 0.001		
M3 COVID-19	-0.49	0.15	0.001		
M4 COVID-19	0.66	0.16	< 0.001		
M5 COVID-19	0.59	0.16	< 0.001		

2.2. Motivation latent change						
Motivation pre-COVID-19	3.1	0.04	< 0.001			
Motivation COVID-19	0					
Motivation change				-0.27	0.05	<
2.3. EC Activities latent change						
EC Activities pre-COVID-19				3.19	0.08	<
EC Activities COVID-19	0					
EC Activities change				-2.08	0.09	<
2.4. Predictor						
Age				9.65	0.16	<
**3. Regression estimates (latent change)**						
Motivation COVID-19 ~ Motivation pre-COVID-19	1					
EC Activities COVID-19 ~ EC Activities pre-COVID-19	1					
**4. Variances**						
4.1. Motivation items						
M1 pre-COVID-19	0.74	0.05	< 0.001			
M2 pre-COVID-19	0.3	0.03	< 0.001			
M3 pre-COVID-19	0.35	0.03	< 0.001			
M4 pre-COVID-19	0.8	0.05	< 0.001			
M5 pre-COVID-19	0.65	0.04	< 0.001			
M1 COVID-19	0.74	0.05	< 0.001			
M2 COVID-19	0.3	0.03	< 0.001			
M3 COVID-19	0.35	0.03	< 0.001			
M4 COVID-19	0.8	0.05	< 0.001			
M5 COVID-19	0.65	0.04	< 0.001			
4.2. Motivation latent change						
Motivation pre-COVID-19				0.59	0.06	<
Motivation COVID-19	0					
Motivation change	0.36	0.05	< 0.001			
4.3. EC Activities latent change						
EC Activities pre-COVID-19				1.13	0.09	<

				9		
EC Activities COVID-19	0					
EC Activities change	1.52	0.09	< 0.001			
4.4. Predictor						
Age				4.56	0.36	<
**5. Co-variances**						
5.1. Latent change						
Motivation pre-COVID-19 ~~ Motivation change	-0.18	0.03	< 0.001			
Motivation change ~~ EC Activities change	0.04	0.02	0.027			
EC Activities change ~~ EC Activities pre-COVID-19	-1.17	0.07	< 0.001			
5.2. Predictors						
EC Activities change ~~ Age	0.10	0.06	0.101			
Motivation change ~~ Age	0.31	0.06	< 0.001			
**6. Correlated error terms**						
M1 pre-COVID-19 ~~ M1 COVID-19	0.08	0.04	0.028			
M2 pre-COVID-19 ~~ M2 COVID-19	0.07	0.03	0.005			
M3 pre-COVID-19 ~~ M3 COVID-19	0.13	0.03	< 0.001			
M4 pre-COVID-19 ~~ M4 COVID-19	0.53	0.05	< 0.001			
M5 pre-COVID-19 ~~ M5 COVID-19	0.33	0.04	< 0.001			

2. SUPPLEMENTARY DATA

Latent change score models can be replicated using the co-variance matrices of both samples (Table S5).

Table S5. Co-variance matrices (lower diagonal)

**Italy**	***M***															
M1_pre	3.09	1.21														
M2_pre	3.08	0.53	1.12													
M3_pre	3.20	0.42	0.88	1.29												
M4_pre	3.70	0.50	0.71	0.69	1.22											
M5_pre	3.47	0.28	0.67	0.77	0.53	1.19										
M1	2.79	0.28	0.54	0.54	0.48	0.47	1.22									
M2	2.82	0.18	0.67	0.66	0.42	0.60	0.76	1.21								
M3	2.87	0.20	0.68	0.79	0.49	0.59	0.74	0.95	1.27							
M4	3.42	0.29	0.60	0.71	0.78	0.56	0.65	0.69	0.72	1.12						
M5	3.22	0.17	0.48	0.50	0.30	0.80	0.44	0.57	0.49	0.44	1.18					
EC_Act_pre	3.19	0.12	0.06	0.02	0.13	0.12	0.06	-0.03	0.04	0.08	0.20	1.06				
EC_Act	1.12	-0.03	0.08	0.07	0.05	0.08	0.12	0.12	0.11	0.08	0.10	-0.05	0.32			
C_Age	9.65	-0.25	0.10	0.17	-0.16	0.20	0.31	0.27	0.54	0.04	0.51	0.68	0.10	4.57		
C_Gender	0.04	0.04	0.08	0.10	0.01	0.04	0.12	0.12	0.10	0.08	0.04	-0.07	0.04	0.13	0.25	
P_Edu	14.13	0.93	0.30	0.56	1.10	0.33	0.24	-0.08	0.28	0.61	-0.30	0.73	-0.17	-0.59	-0.07	15.09
**Portugal**	***M***															
M1_pre	3.10	1.53														
M2_pre	3.28	0.87	1.48													
M3_pre	3.19	0.89	1.08	1.41												
M4_pre	3.46	0.66	0.82	0.85	1.52											
M5_pre	3.42	0.72	0.97	0.83	0.70	1.33										
M1	3.01	0.63	0.72	0.68	0.37	0.60	1.56									
M2	3.21	0.63	0.93	0.83	0.51	0.73	1.08	1.39								
M3	2.99	0.60	0.76	0.90	0.48	0.67	1.00	1.07	1.32							
M4	3.24	0.53	0.65	0.69	1.01	0.55	0.69	0.76	0.78	1.36						
M5	3.26	0.56	0.78	0.65	0.50	0.91	0.83	0.91	0.83	0.65	1.35					
EC_Act_pre	2.88	0.08	0.09	0.14	0.14	0.10	0.00	-0.02	0.06	0.09	0.02	1.37				
EC_Act	1.29	0.01	0.05	0.05	0.08	-0.01	0.02	0.03	0.06	0.12	0.03	0.16	0.48			
C_Age	10.04	0.01	0.14	0.32	-0.32	0.24	0.29	0.52	0.57	0.06	0.53	0.13	0.10	6.36		
C_Gender	-0.02	0.11	0.08	0.07	0.14	0.08	0.06	0.07	0.07	0.12	0.10	-0.05	0.01	-0.12	0.25	
P_Edu	14.84	0.08	-0.29	-0.06	0.36	-0.31	-0.15	-0.17	-0.03	0.19	-0.26	1.31	0.09	-0.80	0.10	15.42

Note. First column includes variables' mean values; gender: girls = -0.5, boys = 0.5.

3. REFERENCES

Cattell, R. B. (1966). The scree test for the number of factors. *Multivariate Behavioral Research, 1(2)*, 245-276. doi:10.1207/s15327906mbr0102_10

Cheung, G. W., & Rensvold, R. B. (2002). Evaluating goodness-of-fit indexes for testing measurement invariance. *Structural Equation Modeling, 9(2)*, 233-255. doi:10.1207/S15328007SEM0902_5

Revelle, W. (2018). psych: Procedures for personality and psychological research. https://CRAN.R-project.org/package=psyc.

Widaman, K. F., Ferrer, E., & Conger, R. D. (2010). Factorial invariance within longitudinal structural equation models: Measuring the same construct across time. *Child Development Perspectives, 4(1)*, 10-18. doi:10.1111/j.1750-8606.2009.00110.x
